# Silica Nanoparticles vs Nanocapsules from Dye-Stabilized Emulsions: Role of the Comonomer

**DOI:** 10.1021/acs.langmuir.5c01624

**Published:** 2025-06-05

**Authors:** Susanne Sihler, Ulrich Ziener

**Affiliations:** Institute of Organic Chemistry III-Macromolecular Chemistry and Organic Materials, 9189 University of Ulm, Ulm 89081, Germany

## Abstract

Dye-stabilized o/w miniemulsions offer an effective platform for synthesizing silica nano-objects via a sol–gel process at the oil–water interface. The choice of the organotrialkoxysilane precursor has a significant impact on the morphology of the resulting silica structures. When Congo red is used as a stabilizer, narrowly distributed nanoparticles below 30 nm and nanocapsules with diameters under 500 nm are formed. The subtle influence of the selected monomer is linked to the kinetics of the sol–gel process, which depend on factors such as molecular structure, comonomer content, and temperature. Factors that accelerate the sol–gel reaction, such as lower steric demand, higher water solubility of the precursor, and temperature increase, favor capsule formation, while slower reactions tend to produce particles instead.

## Graphical abstract

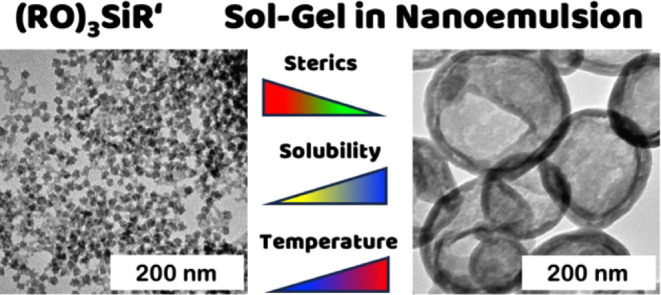

## Introduction

Nature generates a diverse range of complex inorganic structures,^1^ which appear in diatoms, for example, in various forms such as tubes, membranes, and frustules.^2^ These natural architectures have long inspired scientists to replicate and refine them. Silica is one of the most prominent materials in this field, with the particulate morphology on the nanoscale representing the basic form for the development of more complex structures. It dates back to the 1960s, when Stöber’s groundbreaking work on the synthesis of micron-sized, spherical silica particles by the sol–gel process has been published and is still the focus of current research.^3–8^ Many papers have looked at controlling the size and size distribution of silica nanoparticles in the sub-100 nm range, with a focus on monodispersity.^9–16^ In addition to homogeneous solutions as reaction environments, heterophasic systems such as emulsions and microemulsions are also used for the synthesis process.^17–19^ Besides plain spherical nanoparticles, core–shell morphologies,^20^ rod-like shapes,^21^ nonspherical objects,^22^ multicompartment particles,^23^ colloidosomes^24^ or silica capsules have been synthesized.^25^ The latter morphology is preferably formed in heterophase systems like direct^26,27^ or inverse miniemulsions^28,29^ down to hollow spheres with a diameter of 6 nm.^30^ Numerous studies have been carried out on the underlying mechanisms for the formation of different silica structures by the sol–gel process. Polymerization and depolymerization reactions by hydrolysis and condensation lead to monomer–cluster and cluster–cluster aggregation under reaction- or diffusion-limited conditions, which are strongly influenced by the pH value.^31–34^ It has been shown that the sol–gel process can be carried out at the oil–water interface in dye-stabilized miniemulsions^35^ and results in small and narrowly size-distributed nanoparticles and nanocapsules. The silicate structure strongly depends on the choice of stabilizer, i.e., in the presence of negatively charged dyes, nanoparticles are formed, while positively charged dyes lead to nanocapsules.^36^ This is attributed to the different kinetics of the sol–gel process via slow monomer–cluster or fast cluster–cluster aggregation, respectively. As in many sol–gel processes,^31–34^ the differences in kinetics are caused by the strongly different pH, in this case due to the high concentration of negatively and positively charged dye molecules at the oil–water interface.^37^ The dominant influence of the dye molecules is maintained when the silane precursor is varied by partial replacement of tetraethoxysilane (TEOS) with organotrialkoxysilanes. Emulsions stabilized with Congo red (CR) with 1:1 precursor mixtures of octadecyltriethoxysilane (ODTES) or phenyltriethoxysilane (PTES) with TEOS lead exclusively to the formation of nanoparticles.^36^ However, it remains unclear whether this concept also applies to other comonomers, especially those with functional, more polar, and less bulky organo groups.

Here, we report a systematic investigation of the influence of the comonomer structure and content, as well as temperature on the morphology of silicate nano-objects obtained by the sol–gel process in CR-stabilized o/w miniemulsions. A series of 13 different organotrialkoxysilanes have been used in different ratios with TEOS, resulting in nanoparticles or nanocapsules, as shown by transmission electron microscopy (TEM). In certain cases, the morphology of the silicate can be controlled in the direction of particles or capsules by adjusting the monomer composition or temperature alone while retaining the comonomer.

## Experimental Section

### Materials

The dye Congo Red (CR, Merck), the silica precursors tetraethoxysilane (tetraethyl orthosilicate, TEOS, 99.93%, VWR Prolabo), octadecyltriethoxysilane (ODTES, 98%, *n*-isomer 85% min, Alfa Aesar), phenyltriethoxysilane (PTES, >97%, Merck), vinyltriethoxysilane (VTES, 97%, Alfa Aesar), 3-(triethoxysilyl)­propyl methacrylate (MATEOS, >98%, TCI), 3-(methacryloyloxy)­propyltrimethoxysilane (MATMOS, 97%, Alfa Aesar), octadecyltrimethoxysilane (ODTMS, tech. grade, Sigma-Aldrich), 3-mercaptopropyltriethoxysilane (MPTES, 97%, ABCR), *n*-octyltriethoxysilane (OcTES, 97%, Thermo Fisher), trimethoxy­(vinyl)­silane (VTMS, 98%, Sigma-Aldrich), trimethoxyphenylsilane (PTMS, 97%, Sigma-Aldrich), (2-cyanoethyl)­triethoxysilane (CETES, 95%, ABCR), (3-glycidoxypropyl)­triethoxysilane (GPTES, 97%, ABCR), and 3-isocyanatopropyltriethoxysilane (TESPIC, 95%, ABCR), *n*-hexadecane (HD, >98%, TCI), toluene (Tol, VWR), methanol (VWR), and sodium chloride (99.5%, Merck) are used as received. Demineralized water with Milli-Q grade (resistivity: 18 MΩ cm) is used for all experiments.

### Preparation of Emulsions

The aqueous phase (continuous phase, CP) and oil phase (disperse phase, DP) are prepared separately. The CP is prepared by dissolving 5.0 mg of CR in 10 mL of water at pH 6 in a 30 mL screw-cap vessel. The oil phase is prepared in a 2 mL Eppendorf tube by mixing 0.36 mL of silane precursor mixture, 0.18 mL of toluene, and 0.06 mL of HD as an osmotic agent (ultrahydrophobe). DP is added to CP, and the mixture is ultrasonicated with a Branson W450 digital sonifier for 3 min under ice cooling (70% amplitude, 1/4″ tip). For a scale-up to 50 mL, 100 mL, or 200 mL of the aqueous phase with proportionally increased quantities of the other ingredients, a 100, 250, or 500 mL one-neck flask is used instead of the 30 mL screw-cap vessel. The success of emulsion formation is checked by DLS (Figures S1, S2, and Table S1).

### Preparation of Particles or Capsules from Emulsions

Particles or capsules are prepared by either letting the emulsion to stand at room temperature (20 °C) under static conditions or heating it to elevated temperatures in a heating stirrer attachment (“Heat-on”) or an oil bath (for the scale-up experiments) while stirring with a 6 × 15 mm stirring bar (300 rpm) for different periods of time.

### Work Up of the Particles/Capsules

If the particles cannot be collected directly by centrifugation, 3 g of NaCl per 10 mL of emulsion are added. After the complete dissolution of the salt, the mixture is centrifuged (at least 5 min @ at least 6000 rpm). If a pellet forms above the liquid phase, the serum is carefully removed using a syringe and a cannula. The pellet is then carefully washed with water without destroying it and without centrifugation. The particles are then dispersed in MeOH by shaking and in an ultrasonic bath so that the particles are completely dispersed. Finally, they are centrifuged (5 min at 6000 rpm). The washing step with MeOH is repeated until the particles or the MeOH are colorless. In the last washing step before drying, some water is added. If the particles are left in MeOH for longer, some water is also added to prevent the formation of Si–O–Me groups.

### Characterization

#### Transmission Electron Microscopy (TEM)

TEM measurements are performed on a Zeiss EM10 microscope with an acceleration voltage of 120 kV. The samples are prepared by putting 5 μL of the dispersion on a copper grid. They are allowed to dry overnight at room temperature before analyzing them in the microscope.

## Results and Discussion

We have performed the sol–gel method in miniemulsions with hexadecane (HD) as an osmotic agent and Congo red (CR) as a stabilizer at a concentration of 0.5 mg/mL in the continuous phase (CP), unless otherwise stated. It is known that this concentration leads to narrowly distributed nanoparticles with diameters in the range of 5–30 nm when TEOS is used as the silane precursor.^36^ Particle formation via the sol–gel process takes place at the o/w interface by homogeneous nucleation, with the miniemulsion droplets serving as a silica precursor reservoir. A series of organotrialkoxysilanes (RO)_3_SiR′ with and without various functional groups has been employed (Scheme 1). The functional groups have been selected due to their extremely attractive potential uses in subsequent polymerization, addition, or substitution reactions on the silica structures.

**Scheme 1:**
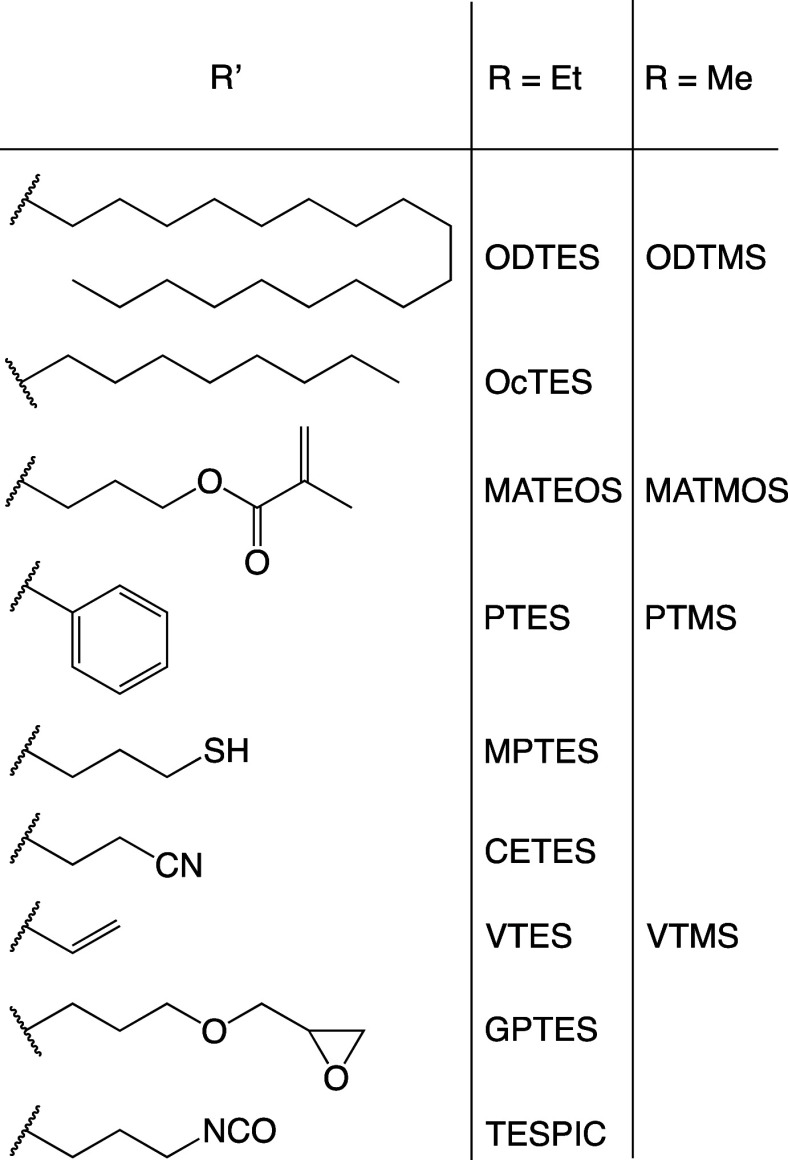
Employed Organotrialkoxysilane Comonomers (RO)_3_SiR′ with Acronyms

For the sol–gel process, the silanes are usually mixed as comonomers with TEOS, as the pure organosilanes often do not provide defined nanostructures. Neither stable emulsions nor defined nanostructures can be obtained with TESPIC. While emulsions with GPTES as a comonomer are sufficiently stable, the sol–gel reaction does not lead to defined silica structures. It is suspected that the functional groups of the two silanes (isocyanate and epoxide) undergo side reactions that destabilize the emulsions or interfere with the sol–gel process. It is assumed that the functional groups hydrolyze rapidly under the basic conditions on the droplet surface created by the negatively charged CR.^36^ The amines and glycols formed in this way increase the water solubility of the silanes and can also lead to cross-linking, which destabilizes the emulsions. Therefore, GPTES and TESPIC are not considered further in the following reactions.

### Variation of Monomer Structure

For comparability, all reactions involving variation of the comonomer structure have been carried out with 50 vol % TEOS and comonomer at room temperature (rt), with the exception of MATEOS and MATMOS. The methacrylates only show defined silica structures at contents of 25 vol % and below. In the case of MATEOS, the sol–gel reaction is rather slow with at least 40 days until no further growth can be observed. Therefore, a higher reaction temperature of 35 °C was selected in order to shorten the reaction time by at least a factor of 2. No influence of the temperature on the morphology is observed for this comonomer. The organotriethoxysilanes ODTES, OcTES, MATEOS, MPTES, and PTES show the formation of nanoparticles with a diameter of about 10–30 nm (Figure 1a–e), as also found with pure TEOS.^36,37^ A closer look at the TEM image of the MPTES system reveals the appearance of particle strands, indicating a tendency toward capsule formation (Figure 1e). This tendency becomes obvious when CETES and VTES are used as comonomers (Figure 1f,g). Here, capsules with diameters of a few tens to 200 nm are clearly formed.

**Figure 1:**
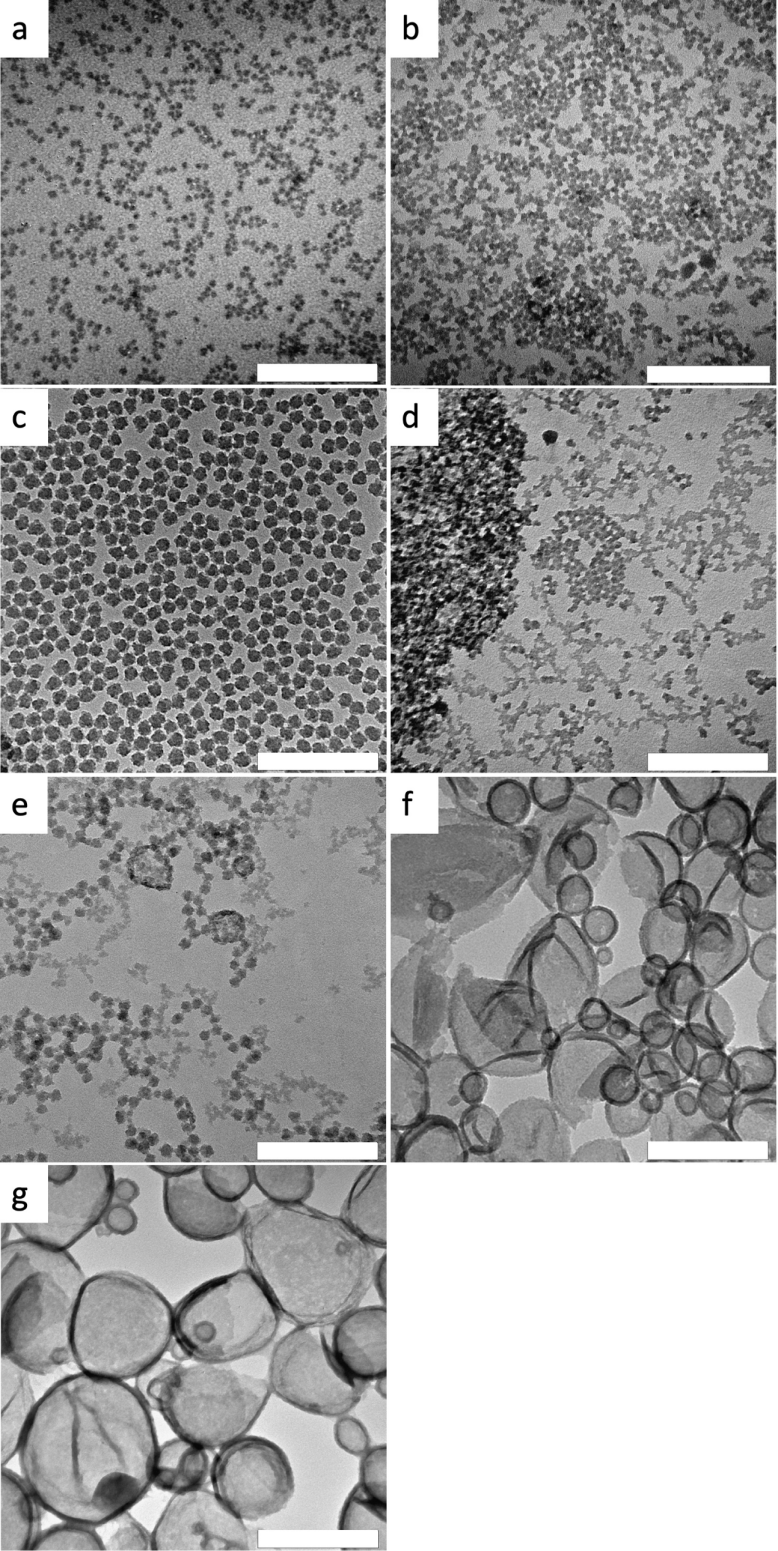
TEM images of silica nano-objects obtained from dye stabilized miniemulsions with precursor mixtures of TEOS and an organotriethoxysilane (50 vol % of comonomer) as the disperse phase at rt with the reaction time in brackets: (a) ODTES (7 d), (b) OcTES (8 d), (c) MATEOS (25 vol %, 35 °C, 20 d), (d) PTES (4 d), (e) MPTES (16 d), (f) CETES (6 d), and (g) VTES (18 d). The scale bar is 200 nm.

The tendency of morphological change due to structural variation of the comonomer becomes even clearer when moving from the triethoxysilanes to the trimethoxysilanes, also at 50 vol %. While ODTMS clearly shows particles (Figure 2a), MATMOS leads to capsules with particulate substructures and aggregates (Figure 2b). For the same reason as with the ethoxy derivative, MATMOS has been used at 25 vol % (see above). At 50 vol % PTMS and VTMS, only capsules with more or less smooth surfaces are observed (Figure 2c,d).

**Figure 2:**
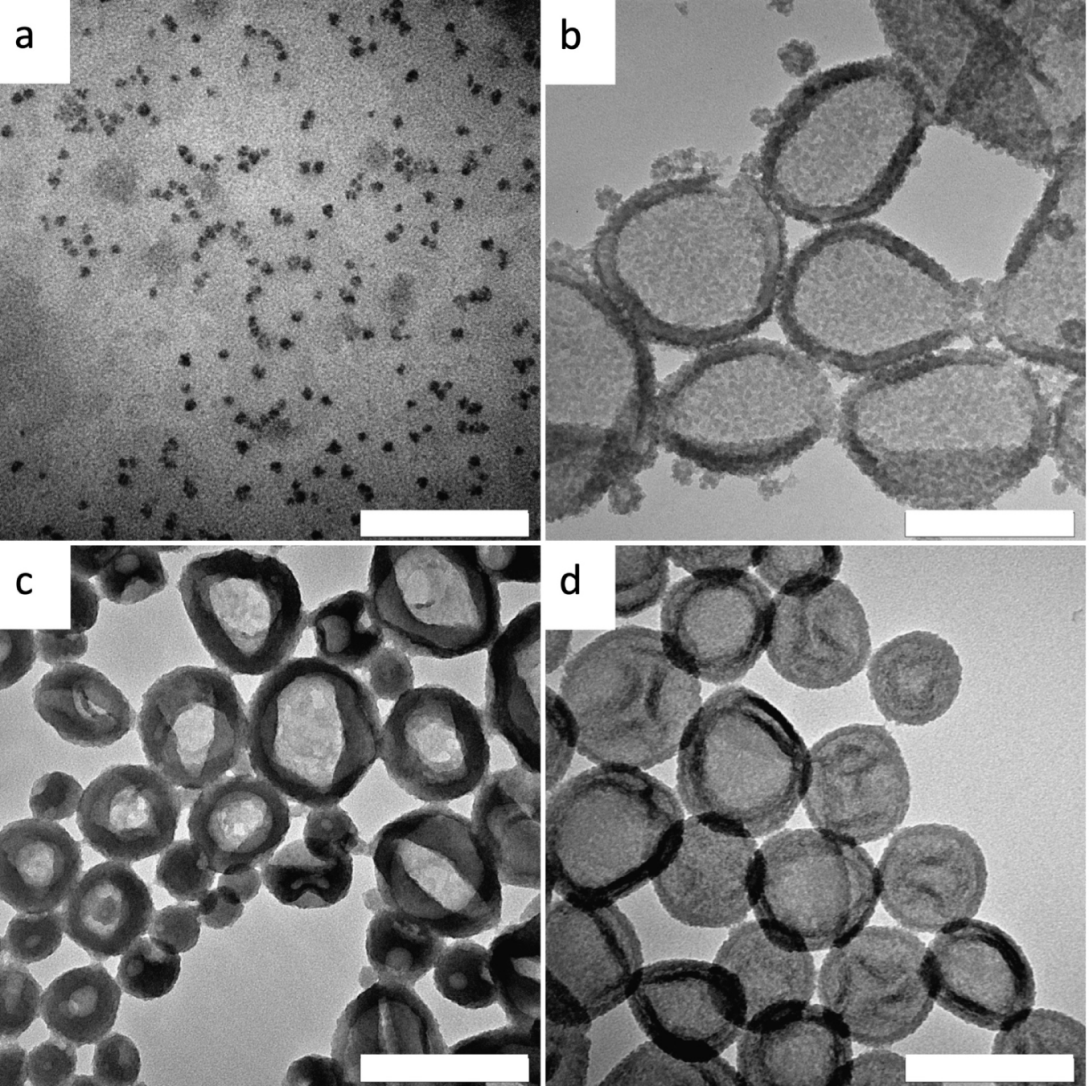
TEM images of silica nano-objects obtained from dye stabilized miniemulsions with precursor mixtures of TEOS and an organotrimethoxysilane (50 vol % of comonomer) as the disperse phase at rt with the reaction time in brackets: (a) ODTMS (8 d), (b) MATMOS (25 vol %, 55 d), (c) PTMS (8 d), and (d) VTMS (8 d). The scale bar is 200 nm.

### Variation of Temperature

Although increasing the reaction temperature to 60 °C increases the reaction rate for emulsions that remain stable, the effect on the particle morphology of the silica is only slight for the comonomers ODTES, OcTES, MATEOS, and ODTMS. The size of the particles increases, and the distribution becomes broader, as shown for the comonomer ODTES as an example (see Figure S3). When capsules form at rt, the morphology remains the same even at higher temperatures. The capsules merely increase in wall thickness, as can be seen for CETES as a comonomer (Figure 3a,b). A corresponding behavior is found for PTMS, VTMS, and VTES (see Figure S4), while PTES behaves differently. The emulsions with PTES form particles at rt (see Figure 1d) but capsules in combination with particles are observed at 40 °C. When the temperature is further increased to 60 °C, the particles disappear, and at 80 °C, the wall of the capsules becomes thicker (Figure 3c–f). A similar tendency can be observed with MPTES. While the emulsion destabilizes at 50 vol % at 60 °C, it is stable at 25 vol %, but the formation of capsules is also observed instead of simple particles (see Figure S5).

**Figure 3:**
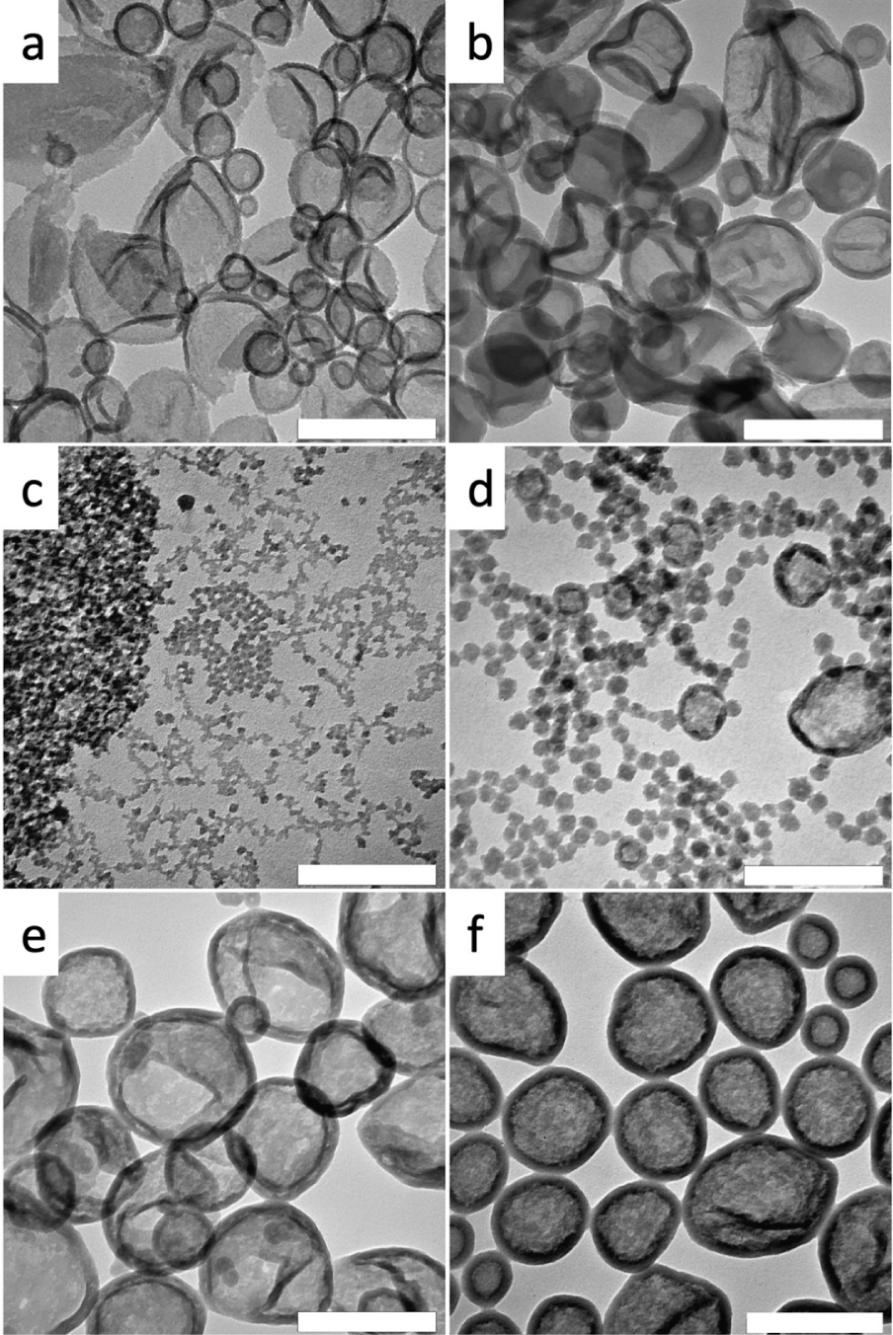
Effect of temperature variation on morphology; 50 vol % CETES (a and b, 6 d reaction time) and 50 vol % PTES (c–f, 4 d reaction time): (a) rt, (b) 60 °C, (c) rt, (d) 40 °C, (e) 60 °C, (f) 80 °C. The scale bar is 200 nm.

### Variation of Comonomer Content

Obviously, not only the temperature can influence the morphology but also the content of the comonomer. We have systematically varied the VTMS content in the dispersed phase from 100 to 11 vol % (Figure 4). Capsules are formed in all cases. At high comonomer content, small capsules with a smooth surface and thick walls are formed, while below 50 vol %, the wall becomes particulate, and between 22 and 11 vol %, the size of the capsules increases significantly, combined with a decrease in wall thickness. The change in wall thickness could be due to a lower density of the sol–gel product due to the steric demand of the vinyl group. Increasing the dye concentration to 2 or 4 mg mL^–1^ has no effect on the morphology but only on the capsule sizes (Figure S6). The latter effect is due to the influence of the stabilizer concentration on the droplet sizes.^35^ The dependence of the wall thickness on the comonomer content is also observed for CETES (Figure S7).

**Figure 4:**
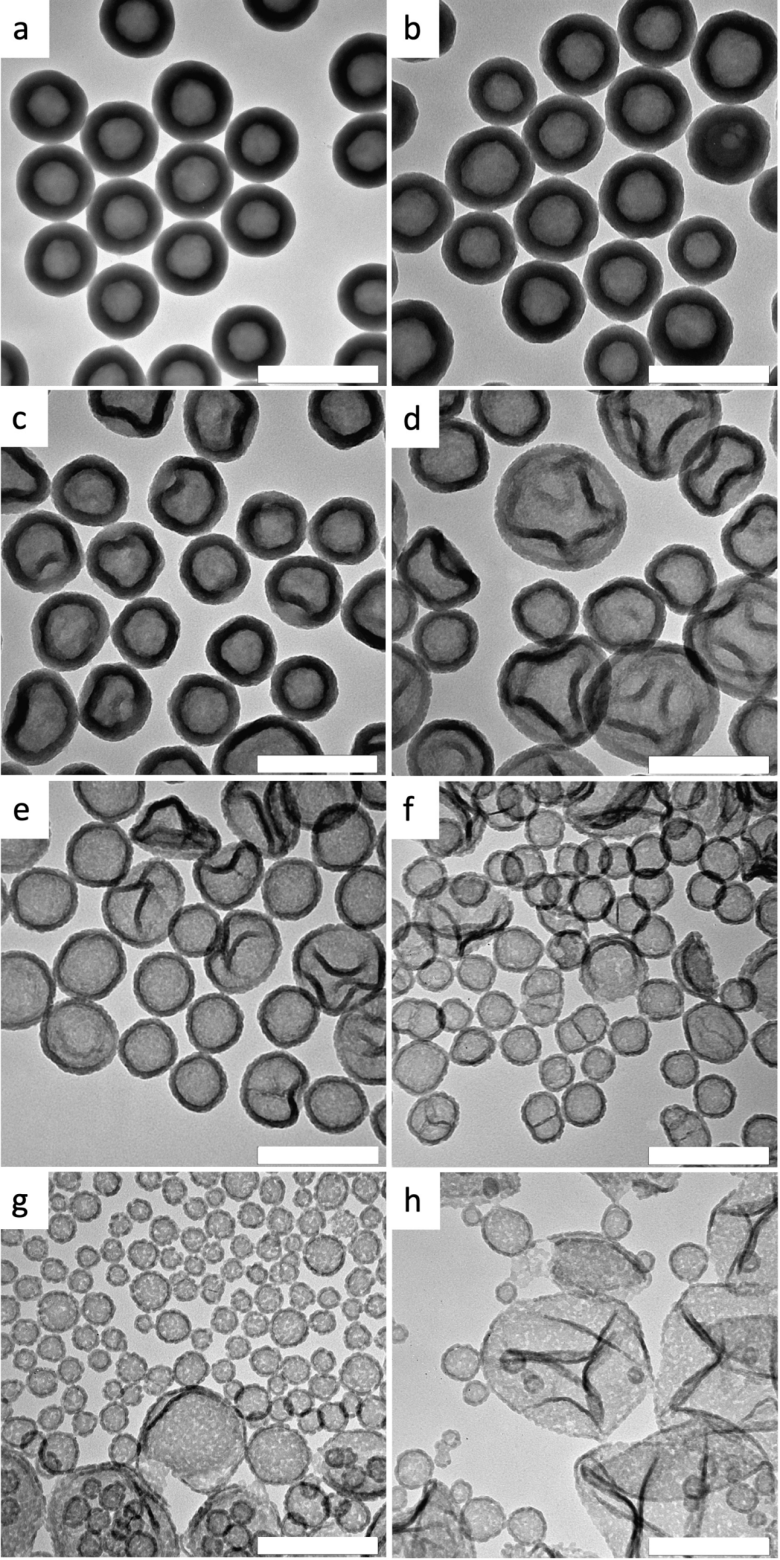
Effect of comonomer content of VTMS on morphology after 6 d reaction time (in vol %): (a) 100, (b) 89, (c) 78, (d) 67, (e) 50 (8 d reaction time), (f) 44, (g) 22, (h) 11. The scale bar is 200 nm.

The morphologies of the silica nanostructures from the sol–gel process of organotrialkoxysilanes with TEOS in dye-stabilized miniemulsions are strongly dependent on various parameters. Since the applied conditions with the negatively charged stabilizer CR for pure TEOS as a precursor lead exclusively to the formation of particles,^36^ the cause of the morphological changes must lie in the presence of the comonomer. As shown above, these changes are strongly influenced by the structure and content of the comonomer, the type of alkoxy group, and the temperature. The reactivity of alkoxysilanes in the sol–gel process, in particular the hydrolysis rate, shows a dependence on the molecular structure and the solubility of the precursor.^38–40^ Alkaline conditions are assumed at the oil/water interface as the reaction site due to the relatively high concentration of negatively charged dye molecules.^36^ Base-catalyzed hydrolysis and condensation rates are increased by electron-withdrawing substituents;^41^ therefore, we assume an influence of steric, inductive, and polarity effects (solubility) on the reaction rates and thus on the resulting morphology. For this reason, the molecular volume *V*_mol_ or log*V*_mol_, respectively, and the octane-water partition coefficient log *P* have been calculated for the various silanes^42^ and are shown together with the experimental data of the chemical shift of the ^29^Si nuclei and the final morphology in Table 1 and Figure 5. Samples with particle or capsule formation independent of the reaction conditions are labeled p and c, respectively, while the emulsions with composition- or temperature-dependent morphologies are shown as p + c.

**Table 1:** Chemical Parameters of the Organotrialkoxysilanes and Resulting Morphology

Sample	*V*_mol_ ^a^	log *P* ^a^	δ^b^	M^c^
ODTES	471.4	9.05	–44.8^d^	p
ODTMS	421.0	8.54	–42.01 ^43^	p
OcTES	303.4	4.80	–44.8 ^44^	p
MATEOS	291.9	2.66	–46.1 ^44^	p
MATMOS	241.5	1.54	–42.6 ^44^	p + c
PTES	240.6	3.04	–57.9 ^45^	p + c
MPTES	237.3	1.93	–45.88 ^46^	p + c
CETES	219.7	0.80	–49.8 ^47^	c
VTES	197.0	1.64	–58.7 ^44^	c
PTMS	190.2	1.91	–55.1 ^48^	c
VTMS	146.6	0.51	–55.4 ^44^	c

a Molecular volume *V*_mol_ in Å^3^ and logarithmic partition coefficient log *P*, both calculated with Molinspiration Cheminformatics free web services.^42^

b Chemical shift of ^29^Si in ppm.

c Morphology: p = particle, c = capsule.

d Value estimated from the experimental data of the alkyltriethoxysilanes with hexyl (−44.86 ppm),^49^ octyl (−44.8 ppm),^44^ and dodecyl chains (−44.84 ppm).^49^

**Figure 5:**
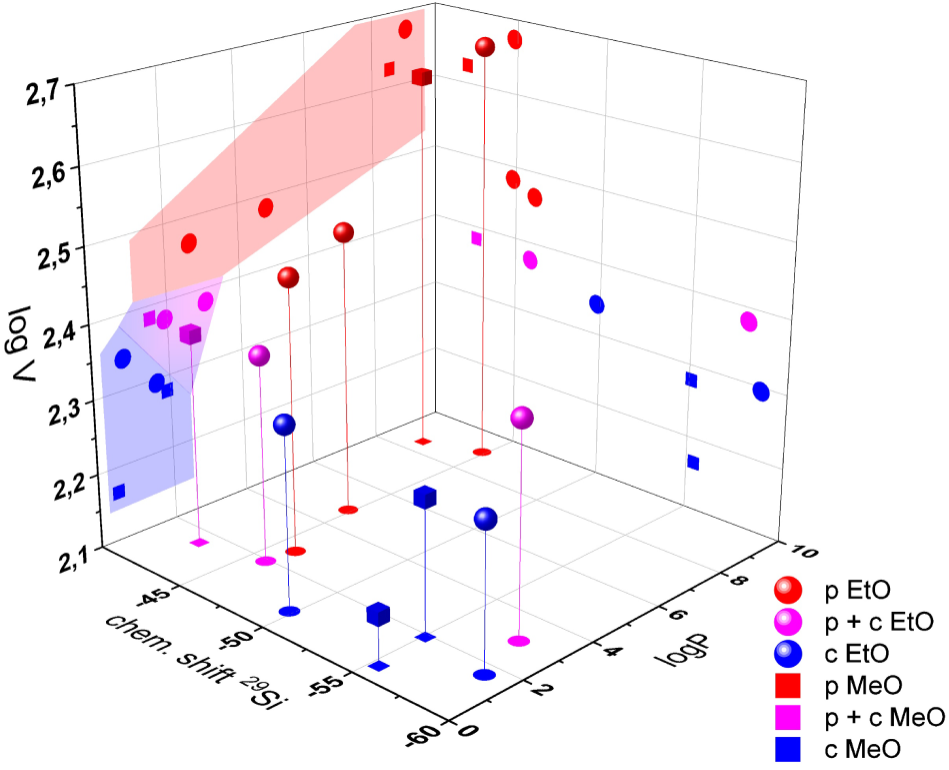
Steric, electronic and polarity effects on the resulting morphology for the organotrialkoxysilanes with ethoxy (EtO) or methoxy (MeO) groups: red: particles (p), blue: capsules (c), pink: particles and capsules (p + c).

*V*_mol_ is considered a measure of the steric demand of organotrialkoxysilanes during the nucleophilic attack in the hydrolysis and condensation reaction steps. log *P* serves as an indicator of the lipophilicity and solubility within the hydrophobic droplets of the emulsion. The chemical shift provides information about the electron density at the silicon center and thus reflects the inductive effects on reactivity. It can be observed that greater steric demand and higher oil solubility favor particle formation over capsule formation and vice versa. Moreover, there appears to be a clear correlation between the chemical shift δ of the ^29^Si nuclei and the resulting morphology: capsule formation is associated with relatively high-field shifts, whereas particle formation corresponds to low-field shifts. However, this correlation is less pronounced than that for the previous parameters, as indicated by the broad range of δ values between −42.6 ppm (MATMOS) and −57.9 ppm (PTES) (Figure 5). More importantly, a lower δ value, which corresponds to higher electron density and lower reactivity for nucleophilic attack, contradicts the experimental observations. Therefore, inductive effects can be considered less significant for the reactivity of the different silanes and the resulting morphology of the silica species. Previous studies have demonstrated that the choice of stabilizing dye significantly influences the sol–gel process and, consequently, the resulting morphology. Hydrolysis has been identified as the rate-determining step, proceeding much faster in the case of capsule formation, which follows a cluster–cluster aggregation mechanism, than in emulsions forming particles via monomer–cluster growth.^36,37^ We propose that in this study, the hydrolysis rate of the silanes at the oil–water interface also determines the final morphology of the silica structures. This is consistent with the increased accessibility of silanes to the reaction sites (interface) and reactants (OH^–^, SiO^–^) at the silicon center, which is enhanced by lower steric demand of both the organic substituent and alkoxy group (smaller log *V*_mol_) and by higher water solubility (smaller log *P*). Additionally, an increase in temperature accelerates hydrolysis and promotes capsule formation. However, the effect of temperature is only significant when the system is near a phase transition, as seen for the comonomers MATMOS, MPTES, and PTES (Table 1 and Figure 5). For the other comonomers, the morphology remains unchanged, with only the particle size and distribution being affected.

## Conclusion

Miniemulsions stabilized with Congo red can be utilized for the synthesis of silica nano-objects via a sol–gel process. For this purpose, various organotrialkoxysilanes have been used as comonomers alongside tetraethoxysilane (TEOS). Depending on the structure of the comonomer, either small, narrowly distributed nanoparticles or capsules are formed. By adjusting the comonomer content or the temperature, the morphology can be shifted from particles to capsules and vice versa. The precise control of morphology is attributed to the influence of these parameters on the kinetics of the sol–gel process at the oil–water interface. Specifically, factors that accelerate hydrolysis, such as lower steric demand and higher water solubility, favor capsule formation. This fine-tuning enables the creation of capsules with presumably porous shells, similar to colloidosomes. Beyond morphological control, the introduced functional groups allow for further chemical modification and adaptation to specific applications. For instance, capsules can serve as carriers, while particles can act as markers, particularly in biomedical applications. Successful functionalization requires the efficient incorporation of organo groups into the silica structures. While this is straightforward for pure organotrialkoxysilanes and evident for capsule-forming comonomers, it is less clear for particle-preserving comonomers. Nevertheless, as demonstrated in our previous study,^36^ the significant change in the wettability of the silica particles confirms the incorporation of comonomers. Finally, it is worth noting that the synthesis can be easily upscaled by a factor of 20. Moreover, the process eliminates the need for additional solvents or osmotic reagents, making it a highly attractive Green Chemistry approach with significant industrial potential.

## Supplementary Material



## Notes

DLS measurements of o/w miniemulsions with various contents of VTMS, comonomers (Figure S1 and S2); droplet sizes and PDI of miniemulsions with various comonomers and compositions of the disperse phase at rt (Table S1); TEM images from emulsions with OcTES as comonomer (50 vol %) (Figure S3); TEM images from emulsions with various comonomers (Figure S4); TEM images from emulsions with MPTES as comonomer (25 vol %) (Figure S5); TEM images from emulsions with different concentration of stabilizer CR (Figure S6); TEM images from emulsions with varying comonomer content of CETES (Figure S7) (PDF)

## Abbreviations

CETES: (2-cyanoethyl)­triethoxysilane;
CP: continuous phase
CR: Congo red
DLS: dynamic light scattering
DP: disperse phase
GPTES: (3-glycidoxypropyl)­triethoxysilane
HD: *n*-hexadecane
MATEOS: 3-(triethoxysilyl)­propyl methacrylate
MATMOS: 3-(methacryloyloxy)­propyltrimethoxysilane
MPTES: 3-mercaptopropyltriethoxysilane
ODTES: octadecyltriethoxysilane
ODTMS: octadecyltrimethoxysilane
PDI: polydispersity index
PTES: phenyltriethoxysilane
PTMS: phenyltrimethoxysilane
TEM: transmission electron microscopy
TEOS: tetraethoxysilane (tetraethyl orthosilicate)
TESPIC: 3-isocyanatopropyltriethoxysilane
Tol: toluene
VTES: vinyltriethoxysilane
VTMS: vinyltrimethoxysilane
