# Supplementary material for: Silica Nanoparticles vs Nanocapsules from Dye-Stabilized Emulsions: Role of the Comonomer
Source: Langmuir. 2025 Jun 5;41(23):15164–72. doi: 10.1021/acs.langmuir.5c01624 (PMC12177942; doi:10.1021/acs.langmuir.5c01624)
Supplement: Supplementary file 1 [file la5c01624_si_001.pdf]

Supporting Information

Silica nanoparticles vs. nanocapsules from dye stabilized emulsions: Role of the comonomer

*Susanne Sihler, Ulrich Ziener**

Institute of Organic Chemistry III-Macromolecular Chemistry and Organic Materials,

University of Ulm, 89081 Ulm, Germany;

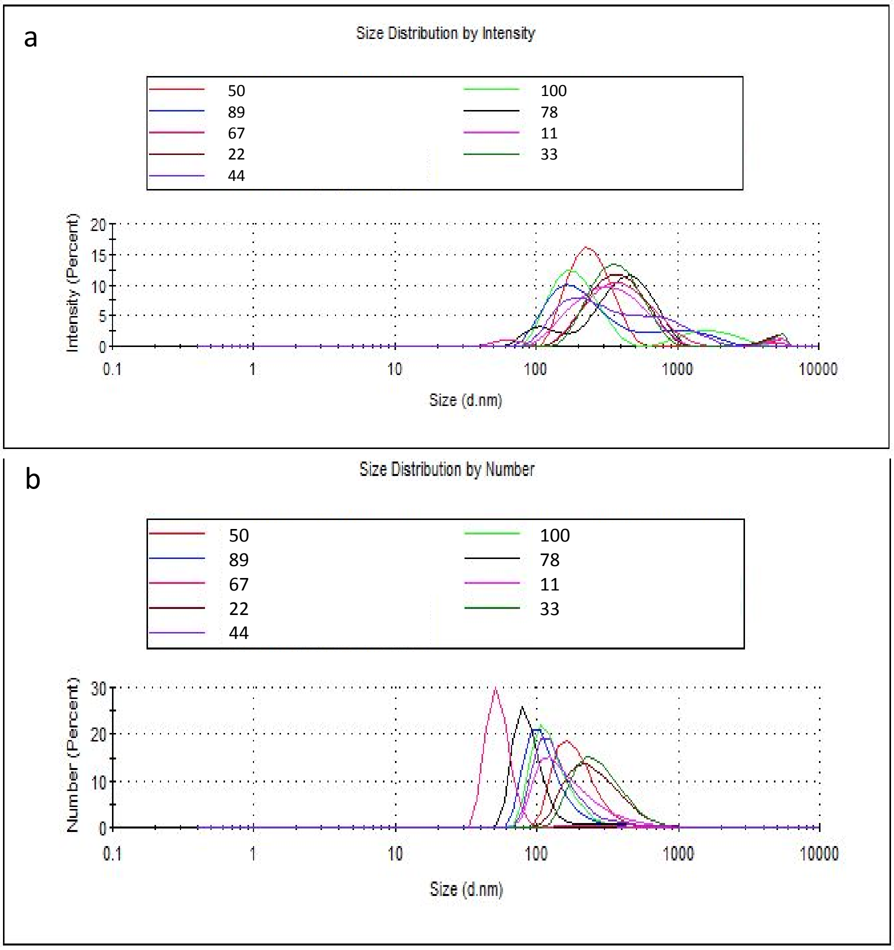

Figure S1. DLS measurements of o/w miniemulsions with various contents of VTMS (in % of the disperse phase, see Table S1) at rt; a) intensity distribution and b) number distribution.

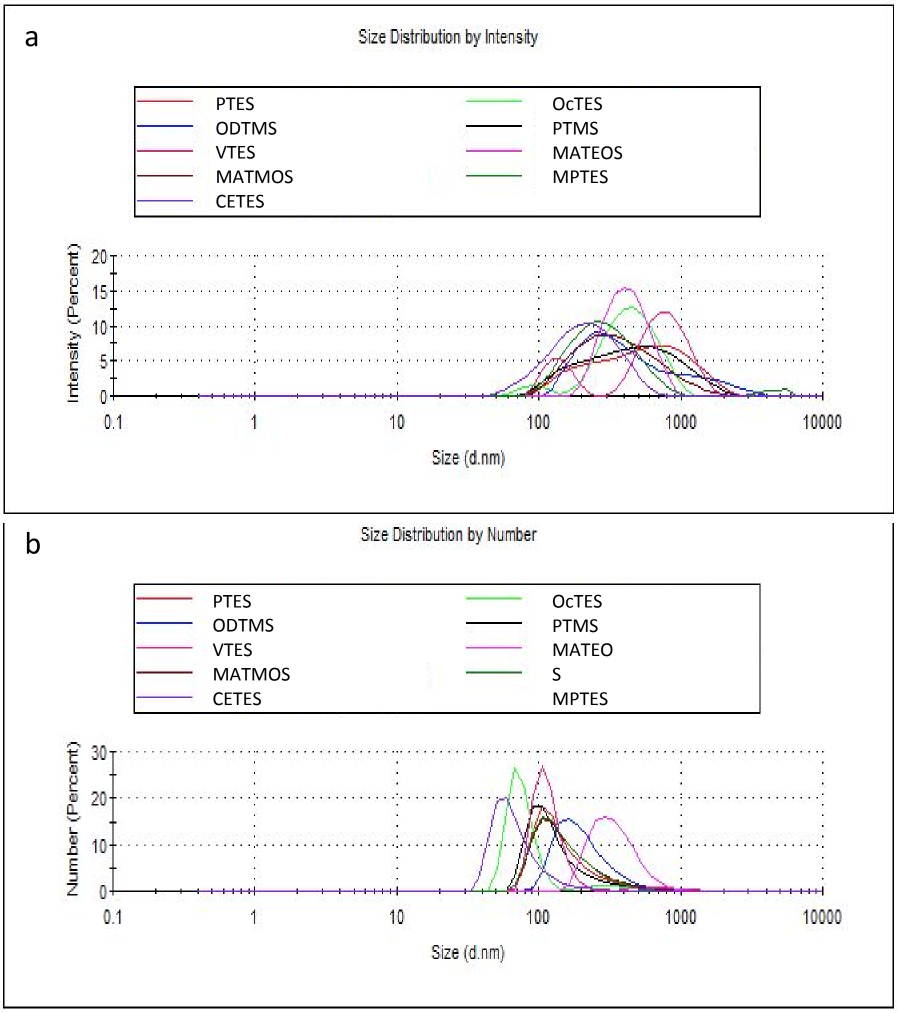

Figure S2. DLS measurements of o/w miniemulsions with various comonomers (50 %, MATMOS 20 %, see Table S1) at rt; a) intensity distribution and b) number distribution.

Table S1. Droplet sizes and PDI of miniemulsions with various comonomers and compositions of the disperse phase at rt. The remaining content to 100 % is TEOS.

**Comonomer**	**Content / %**	**Diameter z-Average / nm**	**PDI**
OcTES	50	356	0.27
MATEOS	50	358	0.185
MATMOS	20	294	0.32
PTES	50	420	0.39
MPTES	50	243	0.22
CETES	50	178	0.22
VTES	50	383	0.42
PTMS	50	326	0.28
VTMS	100	209	0.26
VTMS	89	217	0.28
VTMS	78	290	0.28
VTMS	67	303	0.30
VTMS	50	233	0.21
VTMS	44	310	0.30
VTMS	33	362	0.29
VTMS	22	370	0.32
VTMS	11	322	0.30

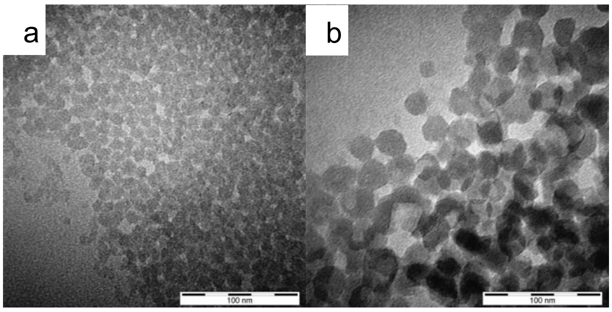

Figure S3. TEM images from emulsions with OcTES as comonomer (50 vol%) after 8d reaction time at a) rt and b) 60 °C reaction temperature.

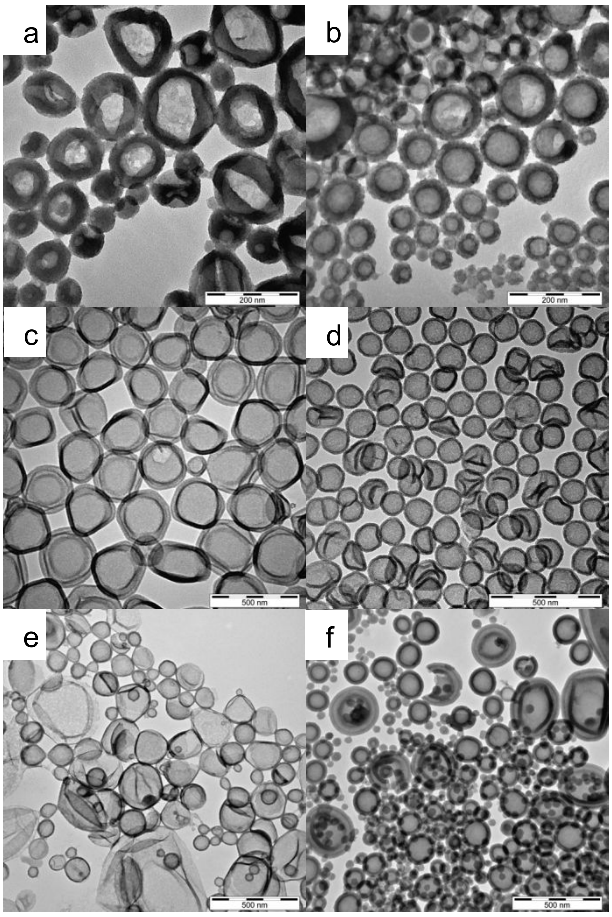

Figure S4. TEM images from emulsions with various comonomers (50 vol%) after 8d (a – d) and 18d (e – f) reaction time: PTMS at a) rt, b) 60 °C; VTMS at c) rt, d) 60 °C; VTES at e) rt, f) 60 °C.

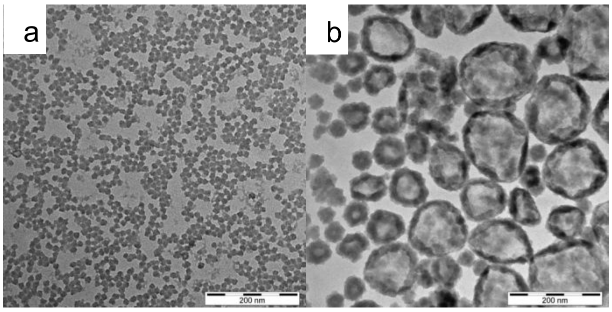

Figure S5. TEM images from emulsions with MPTES as comonomer (25 vol%) after 8d (a) and 5d (b) reaction time at a) rt and b) 60 °C reaction temperature.

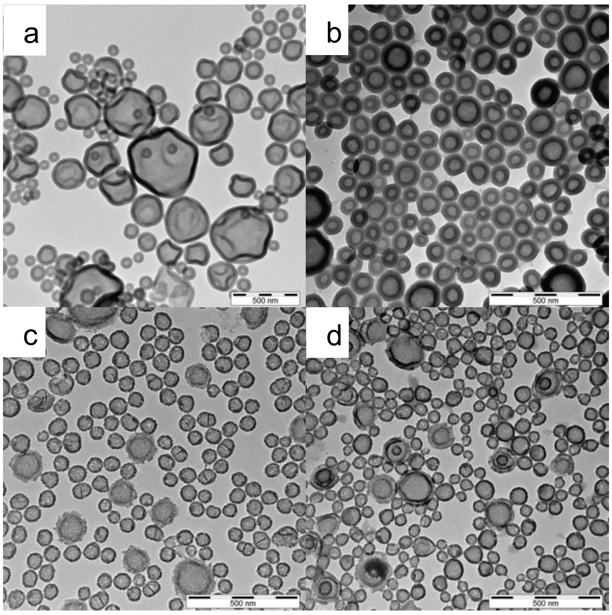

Figure S6. TEM images from emulsions with different concentration of stabilizer CR: a, c) 2 mg mL^{-1}, b, d) 4 mg mL^{-1}) after 5 d reaction time at rt reaction temperature with 100 vol% (a, b) and 50 vol% (c, d) VTMS as comonomer.

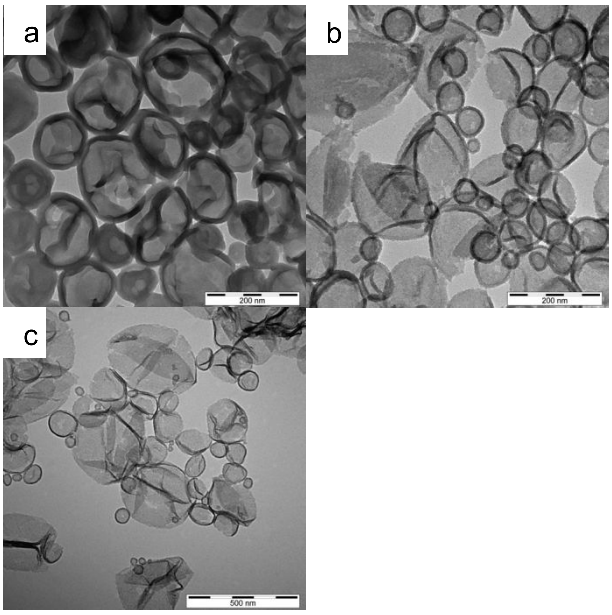

Figure S7. TEM images from emulsions with varying comonomer content of CETES at rt reaction temperature after 6 d (a, b) and 8 d reaction time (c): a) 100 vol%, b) 50 vol%, c) 25 vol%.
